# Cultivating Technical Application Innovation in China’s Vocational Undergraduate Education in the AI Era: A Symbolic Interaction Perspective

**DOI:** 10.3390/bs16091646

**Published:** 2026-09-14

**Authors:** Qiuchen Wu, Bin Bai

**Affiliations:** 1 College of Teacher Education, Ningbo University, Ningbo 315211, China; wuqiuchen@nbu.edu.cn; 2 Faculty of Education, Beijing Normal University, Beijing 100875, China

**Keywords:** technical application innovation, vocational undergraduate education, symbolic interaction theory, artificial intelligence, mixed methods

## Abstract

As artificial intelligence automates routine technical operations, the irreplaceable human contribution shifts toward technical application innovation—making useful improvements within existing technical frameworks through contextual judgment and adaptive problem-solving. China’s vocational undergraduate education system, expanding from 15 pilot institutions in 2019 to 124 by June 2026, is uniquely positioned to cultivate this capability, yet how such innovation is activated through teacher–student interaction remains empirically unexamined. Grounded in symbolic interaction theory, this study employed an exploratory sequential mixed-methods design. A qualitative phase (n = 10) using theory-informed qualitative analysis produced a three-layer account of symbolic transmission, symbolic reception, and interactive mechanisms. A quantitative phase (N = 386) developed a 35-item scale and conducted an initial psychometric evaluation across six institutions. Structural equation modeling showed that interactive mechanisms had the strongest association with technical application innovation capability (β = 0.453), that symbolic reception showed stronger associations with the outcome than symbolic transmission in student self-reports, and that 38–44% of the association between symbolic rules and innovation passed through interactive mechanisms. These patterns are consistent with an innovation-as-emergence-from-interaction framework and provide a starting point for positioning vocational undergraduate education in the AI era.

## 1. Introduction

### 1.1. Technical Work in the AI Era and the Rise in China’s Vocational Undergraduate Education

Since generative AI entered widespread use in late 2022, the scope of automation has expanded steadily—from code generation to engineering design and from industrial quality inspection to equipment fault diagnosis. Operations that can be codified as standardized procedures are being automated systematically (Brynjolfsson et al., 2023). A field experiment with 5179 customer service workers found that generative AI raised productivity among less-skilled workers by an average of 34%, while for high-skilled workers it functioned primarily as an augmentation tool rather than a replacement (Brynjolfsson et al., 2023). Autor (2024) characterized AI’s labor market effects through a substitution–augmentation–creation framework, arguing that the augmentation channel—where AI enables technical workers to engage in more complex, innovative tasks—will fundamentally reshape the objectives of technical education.

What these developments mean for technical workers is this: when AI handles the “follow-the-procedure” tasks, the irreplaceable human contribution shifts to knowing what to do in non-standard situations, spotting opportunities for improvement in existing solutions, and creatively combining knowledge across domains (Dicks et al., 2024). This capability is conceptualized here as technical application innovation—distinct from the scientific or technological invention cultivated in doctoral programs at research universities (Xue & Li, 2022). It centers on making useful improvements within established technical frameworks: optimizing processes, adapting solutions to local conditions, integrating cross-domain approaches, and solving unstructured problems at the point of operation.

Running parallel to the AI revolution is the historic rise in China’s vocational undergraduate education system. In 2019, China launched pilot undergraduate-level vocational programs with just 15 institutions, breaking through the “ceiling” that had confined vocational education to the associate degree level. What followed was a period of extraordinary growth, driven by the 2022 revision of the Vocational Education Law granting vocational undergraduate degrees equal legal standing with conventional university degrees, and the 2024–2035 Education Powerhouse Construction Outline mandating steady expansion of vocational undergraduate institutions and enrollment (Liu & Hardy, 2023). By the end of 2024, there were 51 such institutions; by the end of 2025, 87; and by June 2026, the number had reached 124 (Ministry of Education, 2026). Enrollment surged from 89,900 in 2023 to over 500,000 in 2025, with projections exceeding 600,000 for 2026—a nearly sevenfold increase in three years (Xiong, 2025). Perhaps most tellingly, admission scores at several vocational undergraduate universities now rival those of elite “Double First-Class” institutions: Shenzhen Information Vocational and Technical University reported a minimum admission score of 566 (130 points above the undergraduate cutoff) and Shenzhen Polytechnic University’s physics minimum reached 591 (155 points above the cutoff). Starting salaries for vocational undergraduate graduates are 42% higher than those of associate-degree graduates in the same fields and regions (Ministry of Education, 2026).

Yet scaling up and rising admission scores do not answer a fundamental question: does the instructional model of vocational undergraduate education—particularly its teacher–student interaction patterns—actually match its institutional mission of cultivating technical application innovators? If the pedagogical approach simply extends associate-level technical training to four years without systematically activating students’ capacity for applied innovation through teacher–student interaction, then the expansion represents credential upgrading rather than genuine transformation of the cultivation paradigm.

### 1.2. An Unanswered Question: How Does Innovation Emerge from Interaction?

Existing research on vocational undergraduate education clusters around two themes. One is macro-level institutional design and policy analysis, focused on scaling, legal status, and resource allocation (Liu & Hardy, 2023; Xue & Li, 2022). The other is meso-level curriculum and industry alignment, addressing program design, training facilities, and school-enterprise cooperation (Lu & Wang, 2023). Both traditions rest on an implicit assumption—that high-quality institutional design and resource provision will naturally foster innovation capability—yet this assumption remains untested at the mechanism level. Why do students in the same program, with the same instructors, exhibit systematic differences in innovation performance?

International comparisons render this question more pointed. The German dual system relies on demonstration-imitation relationships between workplace trainers and apprentices (Gessler, 2017). Australia’s competency-based training emphasizes standardized assessment frameworks (Smith, 2010). Nordic models foreground the pedagogical relationship in practical skill formation (Hiim, 2017). Recent scholarship has begun examining innovation cultivation in vocational settings more directly: Fischer and Barabasch (2023) identified interaction as one of five categories promoting creativity in Swiss vocational teacher education. Li et al. (2022) demonstrated through a quasi-experiment that creative thinking instruction outperforms traditional imitation-based methods. G. Wang (2025), in a qualitative study of “order-model” training in China’s construction sector, found that while students gained skill confidence through reformed VET, they still felt pressure to pursue academic degrees due to persistent credentialism—suggesting that adding skills training alone does not address the deeper challenge of cultivating innovation capability. What remains absent from all of these studies is an account of the micro-level symbolic process through which technical application innovation is actually activated in teacher–student encounters.

The central argument of this study is that technical application innovation is not a by-product of technical transmission. It is activated through specific forms of teacher–student interaction. When a student not only learns “this is how the operation is done” but acquires, through interaction, the symbolic tools to understand the logic underlying the technique (decoding), the space to explore alternative approaches within normative boundaries (construction), and the internal drive to make improvement a self-directed habit (internalization), innovation capability begins to emerge (Blumer, 1969). The core mechanism is not transmission but symbolic negotiation (Mead, 1934).

### 1.3. Theoretical Lens: Three Affordances of Symbolic Interaction Theory

Symbolic interaction theory (Mead, 1934; Blumer, 1969) offers a distinctive lens for analyzing this process. Its central proposition—that the meaning of things is assigned, revised, and internalized through social interaction—has three affordances for explaining the emergence of innovation.

First, innovation begins with questioning given meanings. Blumer (1969) argued that people do not passively accept the established meaning of symbols but interpret and revise them based on the situation. In technical education, this implies that innovation does not descend as sudden inspiration—it originates when a student reflects on a standard procedure: “Why must this step be done this way? Is there a better approach?” Stryker’s (1980) structural symbolic interactionism adds that role expectations embedded in social structures both constrain behavior and preserve space for role negotiation—the theoretical basis for the “improving within the bounds of standards” that characterizes technical application innovation.

Second, the space for innovation emerges in the tension between standards and autonomy. In technical education, this tension manifests as a dynamic balance between technical norms (mandatory safety and quality standards) and innovative exploration (testing improvements within acceptable boundaries)—what the qualitative findings identify as the balance mechanism. If normative constraints are too rigid, the space for innovation collapses to zero. If they are too weak, innovation loses its technical grounding. Chen et al. (2021) found that group interaction dynamics constituted one of three emergent themes in the creative process among vocational high school students, lending further support to interaction’s central role.

Third, the internalization of innovation depends on relational processes of symbolic exchange—termed here as relational internalization. Self-determination theory (SDT) describes internalization as the motivational process through which individuals transform external regulations into personal values (Ryan & Deci, 2000). Symbolic interaction theory adds a crucial relational dimension: in vocational education, the internalization of technical symbols is not a private psychological event—it occurs through sustained symbolic exchange between teacher and student. When a teacher, through symbolic regulation (personalized guidance), leads a student to experience technical improvement as “their own project” rather than “the teacher’s requirement,” innovation shifts from an external task to an internal drive. Rahman Riyanda et al. (2022) proposed a new TVET paradigm in which teachers shift from instructors to facilitators—a logic consistent with relational internalization.

### 1.4. Research Questions

This study addresses two sequential research questions:

RQ1 (Qualitative): How is technical application innovation activated through teacher–student symbolic interaction in China’s vocational undergraduate education? What symbolic rules and interactive mechanisms constitute the process architecture of innovation cultivation?

RQ2 (Quantitative): What patterns of association link the symbolic rules and interactive mechanisms identified in the qualitative phase with technical application innovation capability? Do interactive mechanisms mediate the association between symbolic rules and innovation capability?

## 2. Methods

### 2.1. Research Design

This study employed an exploratory sequential mixed-methods design (Creswell & Plano Clark, 2018). The AI context shaped the study in a specific way: as AI increasingly handles routine technical operations, understanding how human-exclusive capabilities—particularly adaptive, context-sensitive innovation—are cultivated becomes an empirical question of growing urgency, not merely a theoretical one. The qualitative phase (January–March 2026) explored the symbolic interaction mechanisms of innovation cultivation through in-depth interviews. The quantitative phase, building on the qualitative findings, developed a psychometric instrument that was pilot-tested (April 2026) and then administered to 386 students across six vocational undergraduate institutions (April–May 2026). The design was chosen for three reasons: the existing literature lacked an adequate theoretical framework, making qualitative exploration a necessary first step (Onwuegbuzie et al., 2010); qualitative findings provided an empirical basis for the content validity of the scale; and the temporal separation of the two phases prevented the qualitative discoveries from being pre-shaped by quantitative hypotheses (Flick, 2018). The qualitative component follows the COREQ 32-item checklist (Tong et al., 2007); the quantitative cross-sectional survey component follows the STROBE guidelines (von Elm et al., 2007).

### 2.2. Qualitative Phase

#### 2.2.1. Methodological Orientation and Theoretical Framework

The analysis was theory-informed rather than purely inductive. Symbolic interaction theory supplied the sensitizing concepts that framed the study (Blumer, 1969), and coding followed the three stages associated with constructivist grounded theory (Charmaz, 2014; Corbin & Strauss, 2015). The three conceptual areas presented below were specified from theory before fieldwork. Coding then elaborated those categories and attached concrete field evidence to them rather than generating them from the data alone.

From the three theoretical affordances discussed above, the analysis attended to three areas: (1) symbolic transmission—how teachers encode technical standards and regulate them into innovation signals, supplying cognitive resources and psychological permission for innovation; (2) symbolic reception—how students decode technical symbols and map them onto innovative practice, forming cognitive maps for innovation and activating them behaviorally; (3) interactive mechanisms—how the cognitive space for innovation is constructed, the tension between standards and exploration is managed, and innovation becomes internalized as part of the student’s agency.

#### 2.2.2. Participants

Participants were selected purposively from three vocational undergraduate institutions in eastern China. The sample comprised six teachers (STEM doctorates, full professors, with at least five years of teaching experience) and four students (junior and senior STEM majors who held provincial-level skills competition awards or national scholarships). The sample favored experienced teachers and academically successful students, which supplied information-rich cases for theory development but also narrowed the range of perspectives represented. Sample size followed the information power principle (Malterud et al., 2016). Saturation was assessed through iterative comparison across the final interviews, though the small number of student participants means this should be treated as preliminary (Hennink & Kaiser, 2022). Participant details appear in Table 1 and Table 2.

#### 2.2.3. Data Collection and Analysis

Fieldwork was conducted from January to March 2026. Each participant took part in one semi-structured in-depth interview (M = 60 min, SD = 8.5, range 48–74 min). All interviews were audio-recorded with written informed consent and transcribed verbatim within 48 h, yielding a corpus of approximately 110,000 Chinese characters. All interviews were conducted in Mandarin Chinese. Interview quotations appearing in this article were translated into English by the first author and independently back-translated by a bilingual researcher not otherwise involved in the study; discrepancies were resolved through discussion. NVivo 14 was used for data management. Two researchers coded independently; Cohen’s Kappa calculated on a randomly selected 30% of the data was 0.84 (95% CI: 0.78–0.90). Disagreements were resolved through discussion, with a third researcher arbitrating the three instances where consensus could not be reached. Coding followed the three-stage procedure (Charmaz, 2014): open coding (combining process coding and in vivo coding; Saldaña, 2021), axial coding (constant comparison; Corbin & Strauss, 2015), and selective coding.

#### 2.2.4. Research Quality

Four strategies were employed to ensure trustworthiness (Lincoln & Guba, 1985): prolonged engagement (12 site visits), peer debriefing (two expert panel sessions), member checking (written confirmation from 8 of 10 participants), and thick description (Geertz, 1973) through rich presentation of original quotations. The first author has a background in vocational education research and entered the study with a theoretical predisposition toward interactionist accounts of learning—this was managed through multi-perspective feedback, systematic search for counter-evidence during coding, and a research journal recording immediate post-interview subjective reactions. 

### 2.3. Quantitative Phase

#### 2.3.1. Core Construct and Hypotheses

Technical Application Innovation Capability (TAIC) was operationalized as the capacity to make useful adaptive improvements within existing technical frameworks, comprising three dimensions (7 items total): (1) contextualized application innovation (3 items)—adapting technical solutions to non-standard working conditions; (2) integrative application innovation (2 items)—creatively combining technical knowledge across domains; (3) optimization-oriented application innovation (2 items)—pursuing continuous incremental improvements to existing processes. This three-dimensional structure captures the cognitive breadth (integration), practical depth (contextualization), and temporal persistence (optimization) of the construct. TAIC overlaps with adaptive performance, creative problem-solving, learning transfer, and innovative work behavior, each of which also describes situated adjustment or improvement. Its intended distinction is narrower and domain-bound: TAIC refers to improvements made within established technical frameworks while respecting operational standards, rather than to novel idea generation or general workplace adaptation. This boundary is examined in the Discussion.

Drawing on the qualitative findings and the theoretical framework, a mediation model was proposed with five hypotheses:

**H1.**  *Symbolic transmission rules are positively associated with TAIC.*

**H2.**  *Symbolic reception rules are positively associated with TAIC.*

**H3.**  *Interactive mechanisms are positively associated with TAIC, and the strength of this association exceeds that of symbolic transmission and reception rules—a hypothesis grounded in symbolic interaction theory’s core expectation that meaning-negotiation processes should carry greater explanatory weight for innovation emergence than either the transmission or reception side alone (Blumer, 1969).*

**H4.**  *The association between symbolic transmission rules and TAIC is partially mediated by interactive mechanisms.*

**H5.**  *The association between symbolic reception rules and TAIC is partially mediated by interactive mechanisms.*

H3’s “stronger association” hypothesis was directly informed by the qualitative finding that multiple teachers and students described interaction as “the core condition for innovation to emerge” (T3, T5, S2).

#### 2.3.2. Instrument

Scale development followed DeVellis’ (2021) eight-step procedure. Items were deductively generated from the three-level qualitative coding structure, with an item-coding traceability matrix documenting the qualitative origin of every item. The resulting Vocational Undergraduate Technical Application Innovation Scale comprises 35 items across ten latent dimensions, all rated on a 5-point Likert scale (1 = strongly disagree, 5 = strongly agree). Complete item wording appears in Appendix A.

Symbolic Transmission Scale (STS, 8 items, α = 0.826): Technical Standard Setting (TSS, 4 items, traced to codes A1–A2) and Personalized Guidance (PG, 4 items, traced to A3–A4). Example: “When explaining technical standards, the teacher explains the design logic behind them, not just the operational steps” (TSS3).

Symbolic Reception Scale (SRS, 8 items, α = 0.823): Technical Understanding (TU, 4 items, traced to A5–A7) and Technical Practice (TP, 4 items, traced to A8–A9). Example: “Based on my understanding of technical principles, I can reflect on whether operational steps could be improved” (TU4).

Interactive Mechanism Scale (IMS, 12 items, α = 0.879): Construction Mechanism (CM, 4 items, traced to A10–A11), Balance Mechanism (BM, 4 items, traced to A12–A13), and Internalization Mechanism (IM, 4 items, traced to A14–A15). Examples: “Discussions with the teacher often give me ideas for improving existing technical approaches” (CM2); “The teacher strictly requires me to follow operational standards while also encouraging me to think about whether there is a better way” (BM2).

Technical Application Innovation Capability Scale (TAIC, 7 items, α = 0.859): Contextualized innovation (3 items), integrative innovation (2 items), and optimization-oriented innovation (2 items). Example: “When facing non-standard technical problems, I can propose adaptive solutions based on my understanding of technical principles” (TAIC1).

The TAIC scale was developed for this study. While the development followed rigorous procedures and the instrument shows strong initial psychometric properties, two caveats apply: (a) the EFA and CFA were conducted on split halves of a single administration wave (split-sample validation), which constitutes internal rather than independent-sample cross-validation; (b) convergent validity with objective innovation output indicators (e.g., patents, technical improvement projects, competition performance) remains to be established—these are matters for subsequent research (see Section 4.4).

#### 2.3.3. Content Validity and Pilot Testing

Five experts in education and psychology (from four different universities, all with English-language SSCI publication records) completed two rounds of independent ratings. Round 2 achieved S-CVI/Ave = 0.94, with all item-level I-CVI values ≥ 0.80 and Fleiss’ Kappa = 0.76. Pilot testing was conducted in April 2026 (n = 62). Item analysis showed significant critical ratios for all items (*p* < 0.01), with corrected item-total correlations ranging from 0.46 to 0.78. The EFA was used for item screening rather than structural claims, because a sample of 62 respondents is too small for a stable ten-factor solution. All 35 items were retained for the main administration.

#### 2.3.4. Main Administration and Sample

The main survey was conducted in April–May 2026. A stratified cluster sampling design was used, stratifying by region—East (Jiangsu, Zhejiang), Central (Hubei, Hunan), and West (Sichuan, Shaanxi)—and clustering by institution. Six vocational undergraduate institutions were selected, from which junior and senior STEM students were sampled. The 35 items were presented in four blocks corresponding to the four scales (STS, SRS, IMS, TAIC), with item order randomized within each block to mitigate order effects; the block order was fixed for all respondents. Of 420 questionnaires distributed, 386 valid responses were returned (91.9% effective response rate). Little’s MCAR test supported the assumption of completely random missingness (χ^2^ = 156.32, df = 142, *p* = 0.190); missing values were imputed using the EM algorithm.

The full sample was randomly split into an exploratory subsample A (n_1_ = 193, EFA) and a confirmatory subsample B (n_2_ = 193, CFA) using SPSS 27.0’s random number generator. The two subsamples showed no significant differences in gender, grade, major, or regional distribution (all χ^2^ tests *p* > 0.05). The full sample (N = 386) was used for SEM analysis. Sample characteristics appear in Table 3.

#### 2.3.5. Data Analysis Strategy

Subsample A (n_1_ = 193): principal axis factoring EFA with Promax oblique rotation (κ = 4). Factor retention was determined by the convergence of eigenvalues > 1.0, scree plot inspection, and parallel analysis based on 95th-percentile random data eigenvalues. Subsample B (n_2_ = 193): CFA (Mplus 8.8, MLR estimation), with discriminant validity assessed via HTMT ratios (criterion < 0.85; Henseler et al., 2015). Full sample (N = 386): SEM with MLR estimation; indirect effects tested via bootstrap (5000 draws, bias-corrected 95% CI; Preacher & Hayes, 2008). Model fit was evaluated against conventional thresholds (Kline, 2023): χ^2^/df < 3.0, CFI ≥ 0.90, TLI ≥ 0.90, RMSEA ≤ 0.08, SRMR ≤ 0.08. Effect sizes were interpreted following Cohen (1988).

#### 2.3.6. Common Method Bias Control and Ethics

Procedural controls (Podsakoff et al., 2003) included: emphasis on anonymity and academic purpose in the survey instructions; placement of subscales in separate questionnaire sections with attention-restoration prompts between sections; and a two-minute filler task (digit-symbol substitution) between the first and second halves of the questionnaire. Statistical controls included Harman’s single-factor test, the unmeasured latent method construct (ULMC) approach, and partial correlation analysis with a two-item social desirability marker variable (Steenkamp et al., 2010).

This study was granted an exemption from ethical review by the Ethics Committee of Ningbo University and subsequently conducted data collection. All participants provided written informed consent.

## 3. Results

### 3.1. Qualitative Findings: How Innovation Emerges from Interaction

Three-level coding, organized within the sensitizing dimensions above, produced a three-layer account of the symbolic interaction structure underlying the cultivation of innovation capability (Table 4 and Table 5). The data served mainly to specify how each area operated in this setting. They filled the theory-derived categories with concrete mechanisms and with teachers’ and students’ own accounts, while the overall three-layer structure stayed close to the framework that had guided the study.

#### 3.1.1. Symbolic Transmission: Encoding and Regulation

Technical standard setting as symbolic encoding. How teachers communicated technical standards—whether as rigid instructions emphasizing compliance (“*this must be done this way*”) or as explanations revealing design rationale (“*why it is designed this way*”)—directly shaped whether students acquired the symbolic resources necessary for innovative thinking. T5 articulated this distinction clearly: “*We don’t just want students to know how to do something. We need them to understand why it’s designed that way. Only when they understand the why can they figure things out on their own when they encounter new situations.*” When technical standards were encoded as a symbolic system whose design logic required understanding rather than a checklist of instructions to be followed, what students gained was not merely operational competence but the capacity to reflect on operations—the cognitive precondition for innovation.

Personalized guidance as symbolic regulation of innovation signals. Standardized encoding provided cognitive resources; personalized regulation conveyed something equally important: psychological permission to innovate. When teachers adjusted their guidance strategies based on students’ cognitive readiness, interests, and psychological preparedness, they transmitted a crucial meta-signal—that improvement was welcomed. “*Some students are bolder, they like trying new approaches. You can’t suppress that—you need to help them explore within safe boundaries. But if a student keeps running into difficulties and isn’t meeting targets, you need to adjust. You can’t just keep criticizing*” (T4). This directly illustrates Blumer’s (1969) interpretive process based on situation: innovation signals cannot be transmitted in a standardized fashion—they require symbolically differentiated encoding calibrated to the receiver’s characteristics.

#### 3.1.2. Symbolic Reception: From Understanding to Improving

Technical understanding as symbolic decoding for innovation cognition. Students needed to decode the teacher’s technical symbols into a cognitive map that included space for innovation—a map that answered not only “*how to do it*” but “*where improvement is possible.*” T3 explained: “*Just being able to perform the operation isn’t enough. You need to understand where this operation sits in the whole process. Once you understand the process, you know which links can be varied and which ones absolutely cannot be changed.*” This decoding process exemplifies what Mead (1934) described as taking the role of the other—students interpret the deeper logic of technical symbols by placing themselves in the position of the process designer.

Technical practice as symbolic mapping for innovation action. Decoding provided the cognitive map, but the map needed to be activated in real, non-standard situations. S2, a provincial-level skills competition first-prize winner, described the process precisely: “*During competition preparation, we had the standard procedures down cold. But the equipment at the competition site wasn’t exactly the same as what we practiced on. You have to adjust parameters based on the actual conditions. Our teacher taught us principles, not rigid steps—that’s why I could figure out what adjustments to make.*” The bridge from knowing to doing was critical: “*One of the hardest things in cultivating high-level technical talent is moving from knowing what something is to knowing how to do it—that’s an enormously important step*” (T2).

#### 3.1.3. Interactive Mechanisms: Three Structural Conditions for Innovation

Construction mechanism: generating the cognitive space for innovation. Students were not merely receivers of symbols—they actively constructed their own technical symbol systems through interaction. “*High-level technical talent needs to build their own system. Take programming—everyone’s code has a different style, and that’s their personal understanding coming through*” (T4). The teacher’s role shifted from demonstrator to provider of a flexible framework within which such construction could occur. “*Teacher–student interaction is really important for students’ self-construction. The teacher needs to suggest possible exploration routes based on the student’s situation*” (T5).

Balance mechanism: managing the tension between standards and innovation. Among the three mechanisms, this one was the most instructive for showing how the standards–innovation tension is managed in practice. T3 offered a precise formulation: “Innovation capability is the hardest part. If the teacher is too strict, students only learn to imitate—they don’t learn to think. If the teacher is too loose, that won’t work either—technical operations have safety bottom lines.” The balance mechanism was not a simple compromise between strictness and freedom; rather, it operated by transforming the tension itself into a driver of innovation through institutionalized interaction rules. “In cultivating high-level technical talent, the teacher can’t just produce copies of themselves… High-level technical talent needs to have their own thinking, their own technical characteristics” (T2). Stryker’s (1980) concept of role negotiation finds its concrete expression in technical education through precisely this mechanism.

Internalization mechanism: forming innovative agency. The ultimate marker of innovation cultivation was not a one-time improvement behavior but innovation becoming part of the student’s agency—”I can and should think about how to do this better” becoming an internalized disposition. T6 described the shift: “*Innovation isn’t something the teacher forces out of you. When a student themselves feels ‘I could do this better,’ they’ll actively look for methods. That shift is the most critical thing.*” Two structural conditions supported internalization: goal orientation—“*the technical requirements in every learning activity converge toward the talent cultivation goal*” (T6)—and cyclicality—“*high-level technical talent is a sustainably developing talent group; cultivation objectives need to extend to a longer cycle*” (T2). “*Vocational undergraduate programs can’t just look at companies’ immediate hiring needs and train matching technical personnel. That only produces entry-level talent, and they’ll be severely limited in their later development*” (T5).

### 3.2. Quantitative Findings

#### 3.2.1. Descriptive Statistics and Correlations

Descriptive statistics and Pearson correlations for the full sample (N = 386) appear in Table 6. Cronbach’s α values ranged from 0.823 to 0.879, exceeding the standard for good internal consistency (Nunnally & Bernstein, 1994). Skewness and kurtosis values all fell within ±1.0. Interactive mechanisms showed the strongest correlation with TAIC (r = 0.620), followed by symbolic reception with interactive mechanisms (r = 0.505).

#### 3.2.2. Exploratory and Confirmatory Factor Analysis

Subsample A (n_1_ = 193) EFA. KMO = 0.875, Bartlett’s χ^2^ = 2588.05 (*p* < 0.001). Parallel analysis supported retaining ten factors, corresponding precisely to the ten theoretically expected dimensions (TSS, PG, TU, TP, CM, BM, IM, contextualized innovation, integrative innovation, optimization-oriented innovation). The ten factors explained 64.12% of the cumulative variance. Pattern coefficients ranged from 0.54 to 0.82, with all cross-loadings below 0.30. The three TAIC sub-dimensions each formed distinct factors, supporting the hypothesized multidimensional structure.

Subsample B (n_2_ = 193) CFA. A model with ten first-order factors loading on four higher-order factors (STS, SRS, IMS, TAIC) showed good fit: χ^2^(557) = 986.12, χ^2^/df = 1.77, CFI = 0.94, TLI = 0.93, RMSEA = 0.045 (90% CI: 0.037–0.053), SRMR = 0.050. Standardized loadings ranged from 0.56 to 0.83 (*p* < 0.001). This four-factor model fit better than alternative models in which the items loaded on fewer higher-order factors (ΔCFI ≥ 0.05; Cheung & Rensvold, 2002). Composite reliability ranged from 0.83 to 0.90 and AVE from 0.54 to 0.66 (Fornell & Larcker, 1981). HTMT ratios ranged from 0.55 to 0.83. The highest value, 0.83, approached the 0.85 threshold, so discriminant validity should be treated as acceptable rather than strong (Henseler et al., 2015).

#### 3.2.3. Common Method Bias

In Harman’s single-factor test, the first unrotated factor accounted for 28.43% of variance (<40% threshold). ULMC model fit improvement was negligible (ΔCFI = 0.01). Partial correlations controlling for the marker variable changed by less than 0.06; all significant correlations were preserved. VIF values ranged from 1.32 to 2.14. Taken together, these four checks indicate that CMB did not pose a serious threat (Podsakoff et al., 2003).

#### 3.2.4. Structural Model and Hypothesis Testing

The full-sample SEM showed adequate fit: χ^2^(559) = 1073.19, χ^2^/df = 1.92, CFI = 0.92, TLI = 0.91, RMSEA = 0.049 (90% CI: 0.042–0.056), SRMR = 0.052 (Hu & Bentler, 1999).

Direct effects are reported in Table 7. H1 (β = 0.111, *p* = 0.028), H2 (β = 0.261, *p* < 0.001), and H3 (β = 0.453, *p* < 0.001) were all supported. Although H1 reached statistical significance, its standardized coefficient (β = 0.111) was small, so this association should be read as modest. The direct association between interactive mechanisms and TAIC was significantly stronger than that of symbolic transmission rules (Δβ = 0.342, *p* < 0.001) and symbolic reception rules (Δβ = 0.192, *p* = 0.009). The path from symbolic reception rules to interactive mechanisms (β = 0.450) was approximately three times that from symbolic transmission rules to interactive mechanisms (β = 0.153), consistent with the qualitative finding that “reception is a more important link than transmission” (T5).

The model explained 27.5% of the variance in IMS and 45.8% of the variance in TAIC. Following Cohen’s (1988) benchmarks, R^2^(TAIC) = 0.458 represents a large effect size, indicating that the model accounts for a substantively meaningful proportion of variance in technical application innovation capability.

Indirect effects. Bootstrap results (Table 8) showed that the indirect association of symbolic transmission rules with TAIC through interactive mechanisms was significant (β = 0.069, 95% CI [0.024, 0.124]), accounting for 38.3% of its total standardized coefficient. H4 was supported. The indirect association for symbolic reception rules was substantially larger (β = 0.204, 95% CI [0.098, 0.318]), accounting for 43.9%. H5 was supported. The difference between the two indirect effects was statistically significant (Δβ = 0.135, SE = 0.052, 95% CI [0.038, 0.241]), indicating that symbolic reception rules were more strongly channeled through interactive mechanisms than symbolic transmission rules. The proportional mediation estimates should be interpreted cautiously. They express the indirect effect as a share of the total effect, and when the total effect is small, as for symbolic transmission, the proportion can shift considerably with small changes in either component.

Effect decomposition appears in Table 9. Two patterns stand out. First, the total standardized coefficient for symbolic reception rules (0.465) was 2.6 times that of symbolic transmission rules (0.180)—at the level of statistical association, students’ decoding-mapping processes showed stronger self-reported associations than teachers’ encoding-regulation. Second, the direct coefficient for interactive mechanisms (0.453) was the single strongest path in the model, consistent with the proposition that innovation is more closely tied to the quality of interaction than to either transmission or reception alone.

### 3.3. Joint Display Integration

A joint display (Fetters et al., 2013) confirmed cross-method consistency at three levels (Table 10): directional alignment (both methods consistently pointed to the centrality of interactive mechanisms), rank-order correspondence (symbolic reception > symbolic transmission in both methods), and mechanistic triangulation (the qualitative “transmission → interaction → internalization” pathway received statistical support in the quantitative mediation analysis).

## 4. Discussion

### 4.1. Innovation as Emergence from Interaction

The central theoretical contribution of this study is the identification of a mechanism-level pathway through which technical application innovation capability is cultivated—termed here as the innovation-as-emergence-from-interaction framework. The quantitative results show that the direct standardized coefficient for interactive mechanisms (0.453) is the highest of any path in the model, and that 38–44% of the association between symbolic rules and innovation capability passes through interactive mechanisms—a pattern consistent with the proposition that innovation is shaped through interaction rather than delivered through instruction. The qualitative findings reveal why: the construction mechanism provides the cognitive space for innovation (the flexible framework), the balance mechanism manages the normative tension between standards and exploration (institutionalized interaction rules), and the internalization mechanism transforms innovation from an external task into an internal disposition (the shift from “the teacher wants me to improve” to “I can do this better”).

This framework positions itself clearly relative to existing work. Whereas Fischer and Barabasch (2023) identified interaction as one category promoting creativity, the present study further specifies three operative mechanisms through which interaction works and their structural relationships with different symbolic rules. Whereas Li et al. (2022) demonstrated the effectiveness of creative thinking instruction, this study focuses on the type-specificity of technical application innovation—improvement within standards rather than general creativity—which makes the balance mechanism central. Whereas SDT (Ryan & Deci, 2000) frames internalization as an individual motivational process, the present findings add a relational dimension: in vocational education, the internalization of innovative agency occurs through specific patterns of teacher–student symbolic exchange, not as a private psychological event.

### 4.2. Why Reception Outweighs Transmission

The finding that the total standardized coefficient for symbolic reception rules (0.465) is 2.6 times that of symbolic transmission rules (0.180) runs counter to the commonsense expectation that “good teachers make good students.” It is, however, precisely consistent with the core logic of symbolic interaction theory: Blumer (1969) and Mead (1934) insisted that the power to construct meaning lies with the receiver, not the sender. In technical innovation education, students’ innovation potential is more closely tied to how they decode technical symbols—as instructions to be followed versus systems whose logic can be analyzed—and how they map that decoded understanding onto adaptive action in non-standard situations. An alternative reading of this gap warrants attention. Symbolic transmission items ask students to describe what the teacher does, whereas the reception items and the TAIC outcome both ask students to describe their own understanding and behavior. Because the latter two share both a common method and a common self-evaluative source, their association may be partly inflated relative to the transmission path. The checks in Section 3.2.3 address common method variance in general but do not rule out this specific self-concept confound. The 2.6-fold gap should therefore be read as a difference in self-reported associations rather than as direct evidence that reception is theoretically more important than transmission.

This finding carries direct implications for policy. The institutional development of China’s vocational undergraduate education has been concentrated almost entirely on the supply side—teacher training and curriculum standards. The evidence from this study suggests that the balance needs to shift toward the demand side: embedding decoding-strategy training in student learning support systems (e.g., technical roadmap construction, principle-based reflection on standard procedures) and increasing the proportion of non-standard situations in laboratory and practical training settings (e.g., fault diagnosis, cross-brand parameter adaptation, process improvement projects) to systematically strengthen students’ capacity to convert symbolic reception into innovative action.

### 4.3. Institutional Distinctiveness and Implications for the AI Era

Distinguishing vocational from conventional undergraduate education. Conventional undergraduate engineering education is oriented toward theoretical innovation—cultivating the capacity to invent new technical principles—and its instructional interaction is organized around the logic of hypothesis-testing scientific inquiry (Xue & Li, 2022). Vocational undergraduate education, as argued here, should be oriented toward applied innovation—cultivating the capacity to make useful improvements within existing technical frameworks—and its instructional interaction needs to follow the logic of understanding-reflection-improvement. The balance mechanism identified in this study—institutionalized management of the tension between normative constraints and autonomous exploration—is the operational expression of the latter orientation. These two innovation orientations require fundamentally different instructional interaction structures: theoretical innovation requires students to be willing to question fundamental principles (“this theory might be wrong”), while applied innovation requires students to respect technical standards while searching for room for improvement (“this practice could be better”; Chen et al., 2021).

Implications for the AI era. AI’s substitution of technical work follows a clear boundary: any task codifiable as an “if–then” rule will eventually be automated (Brynjolfsson et al., 2023). What AI cannot replace is adaptive judgment in non-standard situations—precisely the core of technical application innovation as defined in this study. Dicks et al. (2024), in a ten-year tracking study of Dutch VET graduates, found that while automation risk depressed starting wages, it did not derail career trajectories—evidence of institutional resilience in vocational education systems that cultivate non-automatable skills. Brynjolfsson et al. (2023) documented the asymmetric effect of generative AI across skill levels—augmenting high-skilled workers’ creative capacity while substituting for routine tasks among less-skilled workers. This asymmetry maps directly onto the two-dimensional structure of the present findings: symbolic transmission (the standardized, codifiable dimension of technical instruction) corresponds to the dimension more susceptible to AI substitution, while interactive mechanisms (non-standardized symbolic negotiation) correspond to the dimension AI cannot easily replicate. The historic opportunity for China’s vocational undergraduate education lies in this distinction: it is positioned to cultivate not the “technical operators” that AI will replace but the “technical application innovators” that AI cannot replace—provided its instructional interaction model shifts from the transmission paradigm toward the construction-balance-internalization mechanisms identified in this study. With 124 institutions and over 600,000 students as of 2026, the urgency of this quality-over-quantity paradigm shift is difficult to overstate.

### 4.4. Limitations and Future Directions

Several limitations warrant attention. First, the qualitative sample was limited to STEM programs at three institutions in eastern China. While this region represents the vanguard of vocational undergraduate development (S. Wang, 2023), applicability to central and western regions and to non-STEM vocational fields (e.g., nursing, early childhood education) requires examination. The qualitative phase also included only four students, all of whom were academically successful, even though the instrument centers on student reception and innovation. Second, the cross-sectional design precludes causal inference—the SEM path coefficients and bootstrap estimates should be interpreted as patterns of association consistent with the hypothesized mediation model, not as evidence of causal pathways (Maxwell & Cole, 2007). The cyclical character of internalization identified in the qualitative findings points toward the need for longitudinal designs. Third, the TAIC scale was developed for this study. While the psychometric properties are strong, the EFA and CFA were based on split halves of a single administration wave (internal validation rather than independent-sample cross-validation; MacCallum et al., 1996), and convergent validity with objective innovation indicators (patents, technical improvement projects, competition outcomes, enterprise innovation performance evaluations) awaits multi-source verification (Dunning et al., 2004). Fourth, innovation capability was measured through self-report. Fifth, the partial mediation pattern (38–44% indirect) suggests unmodeled pathways—organizational innovation climate (Schneider et al., 2013), depth of school-enterprise cooperation (Euler, 2013), or students’ prior technical experience are candidates for future research. Cross-cultural comparison—testing the invariance of the transmission-reception-interaction model across the German dual system, Australian CBT, and Japanese in-company mentoring traditions—is a particularly promising direction. A further limitation is that students were nested within six institutions while the structural model treated responses as independent, because the number of clusters was too small for multilevel modeling. Unmodeled within-institution dependence could affect the standard errors, particularly for the small H1 coefficient. A related point concerns the qualitative analysis itself. Because the three organizing areas were carried into the study from symbolic interaction theory, the coding risked confirming the framework it set out to apply, and it was better suited to specifying those areas than to uncovering unexpected categories.

## 5. Conclusions

This study, employing an exploratory sequential mixed-methods design grounded in symbolic interaction theory, examined the cultivation mechanisms of technical application innovation capability in China’s vocational undergraduate education. Three conclusions emerge.

First, interactive mechanisms showed the strongest association with technical application innovation capability (β = 0.453). The three structural mechanisms—construction (generating cognitive space for innovation), balance (managing the standards–innovation tension), and internalization (relational formation of innovative agency)—together accounted for more variance in innovation capability than either transmission or reception alone. This pattern is consistent with the innovation-as-emergence-from-interaction framework.

Second, at the level of statistical association, students’ symbolic reception showed substantially stronger explanatory power than teachers’ symbolic transmission. The total standardized coefficient for reception (0.465) was 2.6 times that of transmission (0.180). This finding points toward the need to rebalance vocational undergraduate education from supply side optimization toward a dual-sided equilibrium—attending as systematically to how students decode and transform technical symbols as to how teachers encode and regulate them.

Third, the institutional value of vocational undergraduate education lies in providing structural conditions for cultivating innovation within normative constraints. The balance mechanism—institutionalized management of the tension between standards adherence and exploratory autonomy—is proposed as the core pedagogical mechanism distinguishing vocational from conventional undergraduate education. This mechanism is consistent with the pattern of demand observed in AI-era labor markets, where non-automatable applied innovation capability commands a growing premium (Brynjolfsson et al., 2023; Autor, 2024).

## Figures and Tables

**Table 1:** Teacher Participants.

Code	Gender	Age	Years Teaching	Specialization	Degree	Title
T1	F	44	10	Intelligent Manufacturing Engineering	PhD	Professor
T2	M	47	17	Robotics Technology	PhD	Professor
T3	M	38	8	Construction Engineering Technology	PhD	Professor
T4	M	41	12	Electronic Information Engineering	PhD	Professor
T5	M	50	23	Electronic Information Engineering	PhD	Professor
T6	F	51	20	Robotics Technology	PhD	Professor

**Table 2:** Student Participants.

Code	Gender	Age	Year	Specialization	Academic Performance
S1	F	21	Junior	Electronic Information Engineering	Provincial Skills Competition, First Prize
S2	M	22	Senior	Robotics Technology	Municipal Skills Competition, Second Prize
S3	M	22	Senior	Robotics Technology	National Scholarship
S4	M	22	Senior	Electronic Information Engineering	Municipal Innovation Competition, Second Prize

**Table 3:** Sample Characteristics (N = 386).

Variable	Category	n	%
Gender	Male/Female	252/134	65.3/34.7
Grade	Junior/Senior	178/208	46.1/53.9
Major	Intelligent Manufacturing Engineering	86	22.3
	Robotics Technology	92	23.8
	Electronic Information Engineering	124	32.1
	Construction Engineering Technology	84	21.8
Region	East/Central/West	156/128/102	40.4/33.2/26.4

**Table 4:** Open Coding Summary.

Code	Label	References	Sources	% of Participants
A1	Logic of technical standard setting	14	8	80
A2	Principles of technical standard setting	15	8	80
A3	Content of personalized guidance	10	9	90
A4	Function of personalized guidance	11	9	90
A5	Capability framework understanding	16	10	100
A6	Risk avoidance understanding	13	9	90
A7	Development planning understanding	15	9	90
A8	Bridging cognition to behavior	18	8	80
A9	Accessing external support for technical practice	19	10	100
A10	Establishing meaning links between symbols and practice	15	8	80
A11	Exploring unconventional paths within flexible frameworks	13	8	80
A12	Dynamic balance between normative constraints and autonomous exploration	11	9	90
A13	Understanding the binary opposition between normative guidance and innovation	13	9	90
A14	Goal-oriented characteristics of sustained internalization	16	10	100
A15	Cyclical characteristics of sustained internalization	14	9	90

**Table 5:** Three-Level Coding Structure.

Selective Code	Axial Code	Open Codes
C1 Symbolic Transmission Rules (Encoding → Regulation)	B1 Technical standard setting as symbolic encoding	A1, A2
	B2 Personalized guidance as symbolic regulation of innovation signals	A3, A4
C2 Symbolic Reception Rules (Decoding → Mapping)	B3 Technical understanding as symbolic decoding for innovation cognition	A5, A6, A7
	B4 Technical practice as symbolic mapping for innovation action	A8, A9
C3 Interactive Mechanisms (Construction → Balance → Internalization)	B5 Construction mechanism: generating the cognitive space for innovation	A10, A11
	B6 Balance mechanism: managing the tension between standards and innovation	A12, A13
	B7 Internalization mechanism: forming innovative agency	A14, A15

**Table 6:** Descriptive Statistics and Pearson Correlation Matrix (N = 386).

Variable	M	SD	Skew	Kurt	α	1	2	3	4
1. STS	3.71	0.68	−0.31	−0.18	0.826	—			
2. SRS	3.58	0.74	−0.31	−0.57	0.823	0.361	—		
3. IMS	3.65	0.71	−0.32	−0.51	0.879	0.315	0.505	—	
4. TAIC	3.60	0.72	−0.28	−0.35	0.859	0.348	0.530	0.620	—

Note: All correlations are significant at *p* < 0.01 (two-tailed). TAIC = Technical Application Innovation Capability.

**Table 7:** Path Coefficients and Hypothesis Testing (N = 386).

Hypothesis	Path	β	SE	t	*p*	Conclusion
H1	STS → TAIC	0.111	0.050	2.20	0.028	Supported
H2	SRS → TAIC	0.261	0.054	4.86	<0.001	Supported
H3	IMS → TAIC	0.453	0.056	8.15	<0.001	Supported
—	STS → IMS	0.153	0.057	2.70	0.007	—
—	SRS → IMS	0.450	0.058	7.78	<0.001	—

**Table 8:** Bootstrap Indirect Effect Estimates (5000 Draws, Bias-Corrected 95% CI).

Path	β	SE	95% CI	% of Total
STS → IMS → TAIC	0.069	0.025	[0.024, 0.124]	38.3
SRS → IMS → TAIC	0.204	0.056	[0.098, 0.318]	43.9

**Table 9:** Decomposition of Effects on TAIC.

Predictor	Direct	Indirect	Total
Symbolic Transmission Rules	0.111	0.069	0.180
Symbolic Reception Rules	0.261	0.204	0.465
Interactive Mechanisms	0.453	—	0.453

**Table 10:** Joint Display of Qualitative and Quantitative Findings.

Construct	Qualitative Finding	Quantitative Evidence	Integrated Interpretation
Symbolic Transmission Rules	Encoding → regulation; cognitive framing effect and psychological permission	Total coefficient = 0.180; 38.3% passes through interaction	Transmission provides necessary but not sufficient conditions for innovation
Symbolic Reception Rules	Decoding → mapping; cognitive innovation map and contextualized adjustment	Total coefficient = 0.465 (2.6× transmission); 43.9% passes through interaction (highest)	Differences in innovation capability are more strongly linked to how students interpret and transform technical symbols than to how teachers present them
Interactive Mechanisms	Construction → balance → internalization; flexible framework, tension management, innovative agency formation	Direct coefficient = 0.453 (strongest); R^2^ (TAIC) = 0.458	Innovation-as-emergence receives strongest quantitative support—interaction quality matters more than either transmission or reception alone

## Data Availability

The data presented in this study are available on request from the corresponding author. The data are not publicly available due to privacy and ethical restrictions.

## Appendix A

All items rated on a 5-point Likert scale (1 = Strongly Disagree, 5 = Strongly Agree).

**Table A1:** Symbolic Transmission Scale (STS, 8 items, α = 0.826).

Item	Dimension	Code Trace	Wording
TSS1	Technical Standard Setting	A1	The teacher provides clear, perceivable expressions of technical standards during technical guidance.
TSS2	Technical Standard Setting	A1	The teacher communicates technical standards through concrete demonstrative behaviors (e.g., progress queries, outcome presentations).
TSS3	Technical Standard Setting	A1, A2	When explaining technical standards, the teacher explains the design logic behind them, not just the operational steps.
TSS4	Technical Standard Setting	A1, A2	The way technical standards are communicated allows me to intuitively grasp the importance of technical norms.
PG1	Personalized Guidance	A3	The teacher adjusts the approach and pace of technical guidance based on my individual situation.
PG2	Personalized Guidance	A3, A4	When I encounter difficulties in learning, the teacher flexibly adjusts the difficulty of milestone targets.
PG3	Personalized Guidance	A3	The teacher takes my cognitive level and learning style into account during guidance.
PG4	Personalized Guidance	A4	The teacher’s personalized guidance makes technical standards more actionable and attainable for me.

**Table A2:** Symbolic Reception Scale (SRS, 8 items, α = 0.823).

Item	Dimension	Code Trace	Wording
TU1	Technical Understanding	A5	I can transform the content of the teacher’s technical guidance into a capability framework for my own development.
TU2	Technical Understanding	A6	I understand the importance of rigid criteria in technical guidance for ensuring operational standardization.
TU3	Technical Understanding	A7	I can establish a phased career development plan based on technical guidance.
TU4	Technical Understanding	A5, A6	Based on my understanding of technical principles, I can reflect on whether operational steps could be improved.
TP1	Technical Practice	A8	I can effectively apply the technical knowledge I have understood to practical operations.
TP2	Technical Practice	A8	I can bridge the conversion channel from technical cognition to technical practice.
TP3	Technical Practice	A9	The laboratory equipment and practice opportunities provided by the school support my technical practice.
TP4	Technical Practice	A8, A9	My performance in technical practice reflects the depth of my understanding of the technical guidance content.

**Table A3:** Interactive Mechanism Scale (IMS, 12 items, α = 0.879).

Item	Dimension	Code Trace	Wording
CM1	Construction Mechanism	A10	Through teacher–student interaction, I can establish meaning links between technical symbols and practice.
CM2	Construction Mechanism	A10, A11	Discussions with the teacher often give me ideas for improving existing technical approaches.
CM3	Construction Mechanism	A10, A11	I can actively construct my own technical cognition system through interaction.
CM4	Construction Mechanism	A11	Autonomous exploration during interaction has helped me form a personalized understanding of technology.
BM1	Balance Mechanism	A12	Normative constraints and autonomous exploration can achieve dynamic balance in technical guidance.
BM2	Balance Mechanism	A12, A13	The teacher strictly requires me to follow operational standards while also encouraging me to think about whether there is a better way.
BM3	Balance Mechanism	A13	I believe that normative guidance and autonomous innovation are complementary, not opposed.
BM4	Balance Mechanism	A12, A13	The interaction process helps me find a balance point between complying with rules and exercising creativity.
IM1	Internalization Mechanism	A14	I can internalize the symbols from technical guidance into components of my own technical capability.
IM2	Internalization Mechanism	A14	Each instance of technical guidance has a clear goal orientation, giving me a sense of direction in learning.
IM3	Internalization Mechanism	A15	The internalization of technical symbols is a gradual accumulation process rather than a one-time event.
IM4	Internalization Mechanism	A15	I recognize that the effects of technical guidance extend beyond the period of schooling into my entire career.

**Table A4:** Technical Application Innovation Capability Scale (TAIC, 7 items, α = 0.859).

Item	Dimension	Code Trace	Wording
TAIC1	Contextualized Application Innovation	A8, A9, A11	When facing non-standard technical problems, I can propose adaptive solutions based on my understanding of technical principles.
TAIC2	Contextualized Application Innovation	A8, A9	When actual equipment or working conditions differ from textbook descriptions, I know how to assess and adjust the operational approach.
TAIC3	Contextualized Application Innovation	A11	When encountering faults or anomalies during practical training, I actively analyze the causes and attempt solutions rather than waiting for the teacher’s instructions.
TAIC4	Integrative Application Innovation	A5, A10	I can creatively integrate technical knowledge from different courses to solve a comprehensive problem.
TAIC5	Integrative Application Innovation	A5, A10	I draw on technical approaches from other domains to address difficulties encountered in my own specialization.
TAIC6	Optimization-Oriented Application Innovation	A11, A14	I actively think about how to optimize existing experimental procedures or operational processes.
TAIC7	Optimization-Oriented Application Innovation	A11, A14	Even when operations comply with standard specifications, I think about whether more efficient or economical approaches exist.

**Table A5:** Scale Summary.

Scale	Items	Dimensions	α	M	SD
STS	TSS1–PG4 (8)	Technical Standard Setting, Personalized Guidance	0.826	3.71	0.68
SRS	TU1–TP4 (8)	Technical Understanding, Technical Practice	0.823	3.58	0.74
IMS	CM1–IM4 (12)	Construction, Balance, Internalization	0.879	3.65	0.71
TAIC	TAIC1–TAIC7 (7)	Contextualized, Integrative, Optimization-Oriented	0.859	3.60	0.72

Note: Code Trace indicates the qualitative open codes (A1–A15 from Table 4) from which each item was deductively derived.
